# Hereditary Hemorrhagic Telangiectasia of the Tongue: A Rare Case Report

**DOI:** 10.1002/ccr3.73883

**Published:** 2026-11-06

**Authors:** Saede Atarbashi‐Moghadam, Ali Lotfi, Fazele Atarbashi‐Moghadam, Mostafa Alam, Milad Baseri, Mohammadreza Kashefi Baher

**Affiliations:** ^1^ Department of Oral and Maxillofacial Pathology School of Dentistry, Shahid Beheshti University of Medical Sciences Tehran Iran; ^2^ Department of Periodontology School of Dentistry, Shahid Beheshti University of Medical Sciences Tehran Iran; ^3^ Department of Oral and Maxillofacial Surgery School of Dentistry, Shahid Beheshti University of Medical Sciences Tehran Iran; ^4^ Health Research Center, Chamran Hospital Tehran Iran

**Keywords:** hereditary hemorrhagic telangiectasia, oral, pathology, tongue

## Abstract

Hereditary hemorrhagic telangiectasia (HHT) is a rare autosomal dominant vascular disorder that may involve the oral mucosa, providing important diagnostic clues for dental practitioners. This report describes a 54‐year‐old female presenting with recurrent bleeding from multiple red‐purple telangiectatic papules on the dorsal surface of the tongue. Her history included recurrent epistaxis, severe iron deficiency anemia, pulmonary involvement, and a positive family history. The lesions blanched on diascopy. Symptomatic management using electrocautery excision under local anesthesia with prophylactic administration of oral amoxicillin (2 g) was performed. Histopathological examination demonstrated thin‐walled vascular spaces containing erythrocytes beneath the epithelium, confirming the diagnosis of HHT. Although postoperative healing was uneventful with immediate bleeding control, recurrence occurred after 1 year, necessitating repeat surgery. This case highlights the progressive and recurrent nature of oral HHT lesions, the importance of early detection, and the need for careful dental management, including antibiotic prophylaxis before invasive procedures associated with a risk of bacteremia in patients with pulmonary involvement.

## Key Clinical Message

Oral hereditary hemorrhagic telangiectasia (HHT) may present as bleeding‐prone lingual papules that pose a diagnostic challenge. Dental practitioners may be the first to recognize HHT and should consider antibiotic prophylaxis for invasive dental procedures that pose a risk of bacteremia in patients with pulmonary involvement.

## 1 Introduction

Hereditary hemorrhagic telangiectasia (HHT), also known as Osler–Weber–Rendu syndrome, is a rare autosomal dominant disorder characterized by abnormal vascular development, with an estimated prevalence of 0.01% to 0.02% [1, 2, 3]. The condition occurs in two main forms: HHT type 1 (HHT1), which is caused by mutations in the endoglin gene, and HHT type 2 (HHT2), which results from mutations in the activin receptor‐like kinase 1 gene [1, 4].

Abnormal vascular structures in HHT are thought to result from dysregulated transforming growth factor‐β (TGF‐β) signaling during vascular development and homeostasis [1]. Vascular lesions range from small mucocutaneous telangiectasias to visceral involvement [1, 3, 4]. In addition, HHT may be associated with arteriovenous malformations (AVMs) affecting the brain, lungs, and liver [2].

The most common clinical manifestation is recurrent epistaxis [1]. The abnormal architecture of small‐ and medium‐sized vessels underlies the clinical manifestations of the disease [5]. Moreover, telangiectasias of the skin and mucosa may occur as part of normal aging but can also be associated with underlying conditions, including liver disease, scleroderma, rosacea, and human immunodeficiency virus (HIV) infection [4].

The vermilion border of the lips is the most commonly affected oral site, although any area of the oral mucosa may be involved [1, 4]. Diagnosis of oral lesions can be challenging, particularly when the clinician is unfamiliar with the characteristic features of telangiectatic lesions [4].

A definite diagnosis of HHT is established when at least three of the following four criteria are present: (a) spontaneous and recurrent epistaxis; (b) mucocutaneous telangiectasias; (c) visceral AVMs (pulmonary, gastrointestinal, hepatic, or cerebral/spinal); and (d) a family history of HHT [1, 3, 6]. The differential diagnosis includes benign liver disease, hereditary benign telangiectasia, CREST (calcinosis, Raynaud's phenomenon, esophageal dysmotility, sclerodactyly, and telangiectasia) syndrome, and ataxia–telangiectasia [1].

The variable clinical manifestations of the disease have led to a range of treatment modalities tailored to individual patients. Although the overall prognosis is favorable, timely diagnosis is essential to prevent complications. Dental practitioners should be aware of HHT, as oral lesions are often the most prominent and readily recognized feature of this syndrome [1].

The aim of this case report is to describe a 54‐year‐old female patient with HHT presenting with multiple complications, including lingual involvement.

## 2 Case History/Examination

A 54‐year‐old female presented to a private dental office (Tehran, Iran) with a chief complaint of recurrent tongue bleeding. The bleeding episodes were predominantly triggered by eating, although spontaneous episodes also occurred.

The patient had been diagnosed with HHT1 at the age of 24, during pregnancy. The diagnosis was established following severe cough, hemoptysis, lung biopsy (pulmonary involvement), and bronchoscopy, based on the clinical diagnostic criteria for HHT. Hemorrhagic lesions gradually appeared on her tongue, nasal mucosa, and skin during her 30s. She reported frequent episodes of epistaxis and a positive family history in her sister and uncle; her two children were unaffected. Notably, she had experienced a similar oral cavity problem 12 years earlier, which had been resolved by minor oral surgery.

The patient suffered from severe iron deficiency anemia, requiring monthly monitoring of complete blood count. When hemoglobin levels fell below 10 g/dL, she received a single dose of intravenous ferric carboxymaltose (Ferinject). Her blood group was O+. Preoperative laboratory tests revealed moderate microcytic hypochromic anemia (Supplementary Table 1).

Clinical examination revealed multiple, variably sized, painless, red‐purple papules on the dorsal surface of the tongue. The lesions measured 3–4 mm, bled easily—approximately three to four times daily—and blanched on diascopy (Figure 1). Tiny red macules were present on the skin without associated discomfort. The remaining oral mucosa was unaffected, and no alterations in tongue sensation were reported.

**FIGURE 1:**
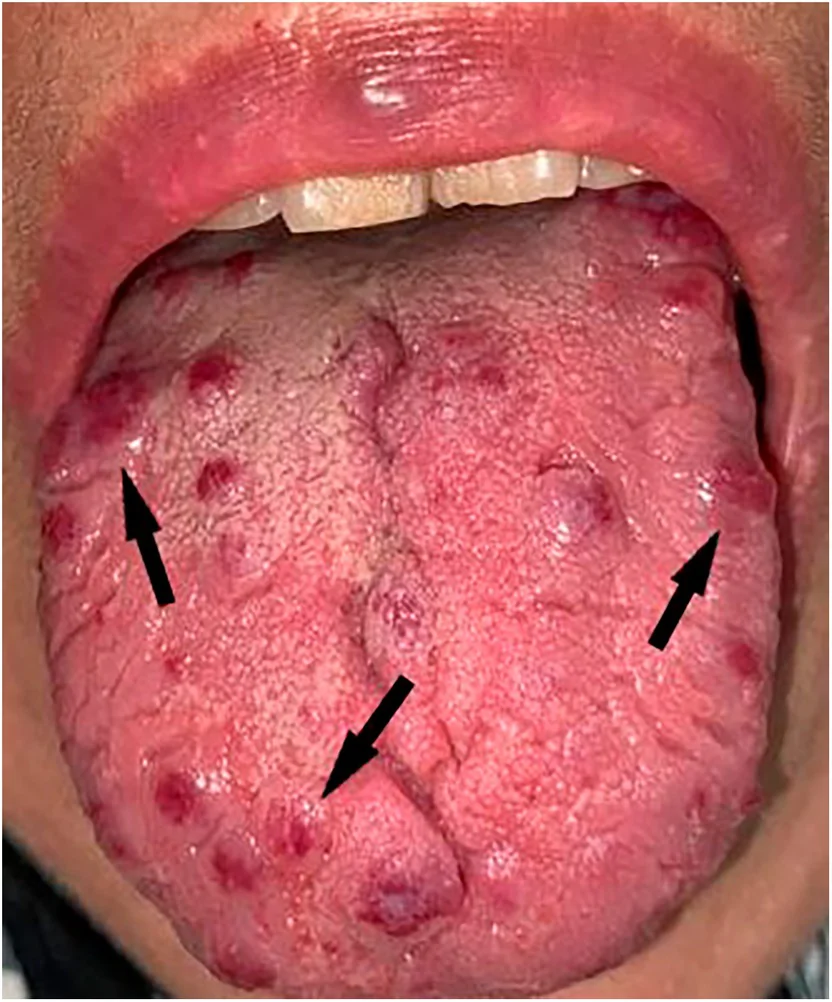
Multiple, varying‐sized, red‐purple papules (arrows) on the dorsal surface of the tongue.

Prior to surgery, prophylactic amoxicillin (2 g, oral route) was administered, and written informed consent was obtained. The larger bleeding papules were ligated with silk sutures and subsequently excised using an electrocautery device under local anesthesia (Figure 2). Postoperative care included amoxicillin 500 mg capsules and 0.2% chlorhexidine gluconate mouthwash.

**FIGURE 2:**
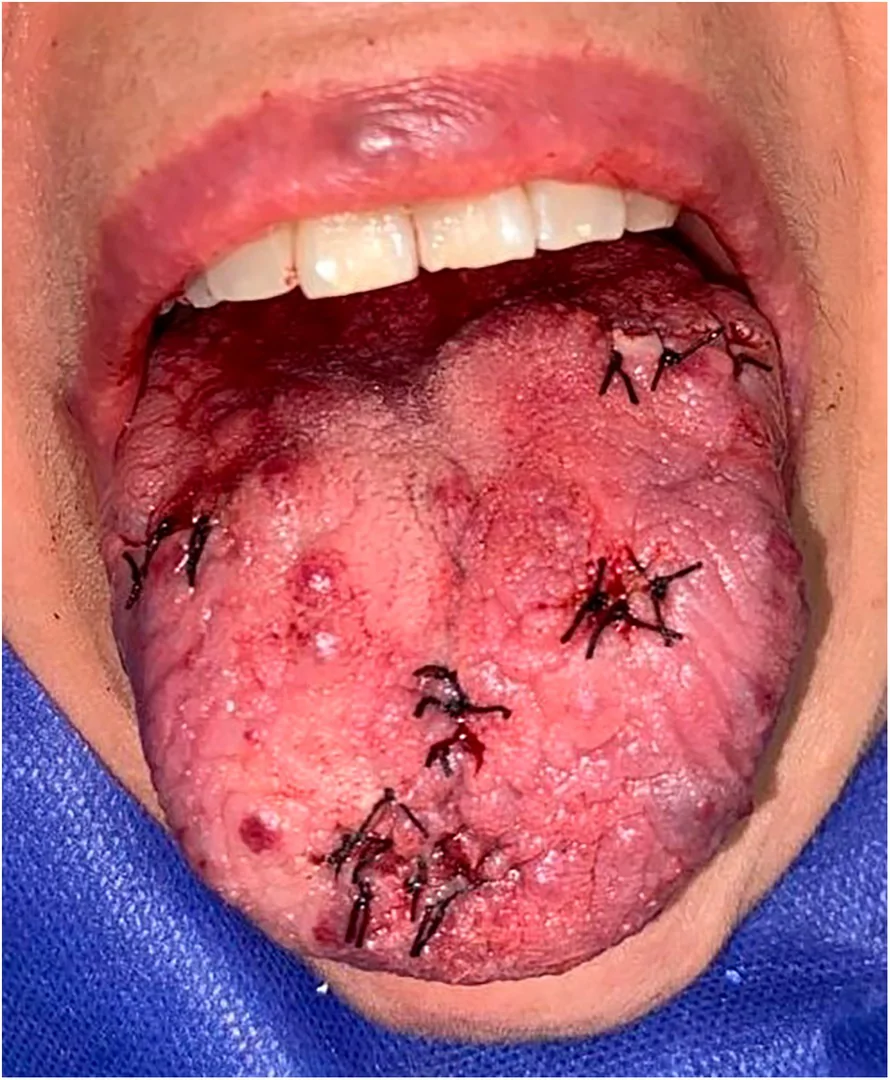
Clinical photograph of the tongue immediately after surgery.

Histopathological examination of the excised specimens revealed thin‐walled vascular spaces containing erythrocytes directly beneath the overlying epithelium, confirming the diagnosis of HHT (Figure 3). At the one‐year follow‐up, the patient returned with the same chief complaint and underwent an identical surgical procedure. Preoperative laboratory values before the second surgery demonstrated mild microcytic hypochromic anemia (Supplementary Table 1). The second surgical intervention served solely as symptomatic management for bothersome tongue bleeding and did not alter the clinical appearance of the tongue.

**FIGURE 3:**
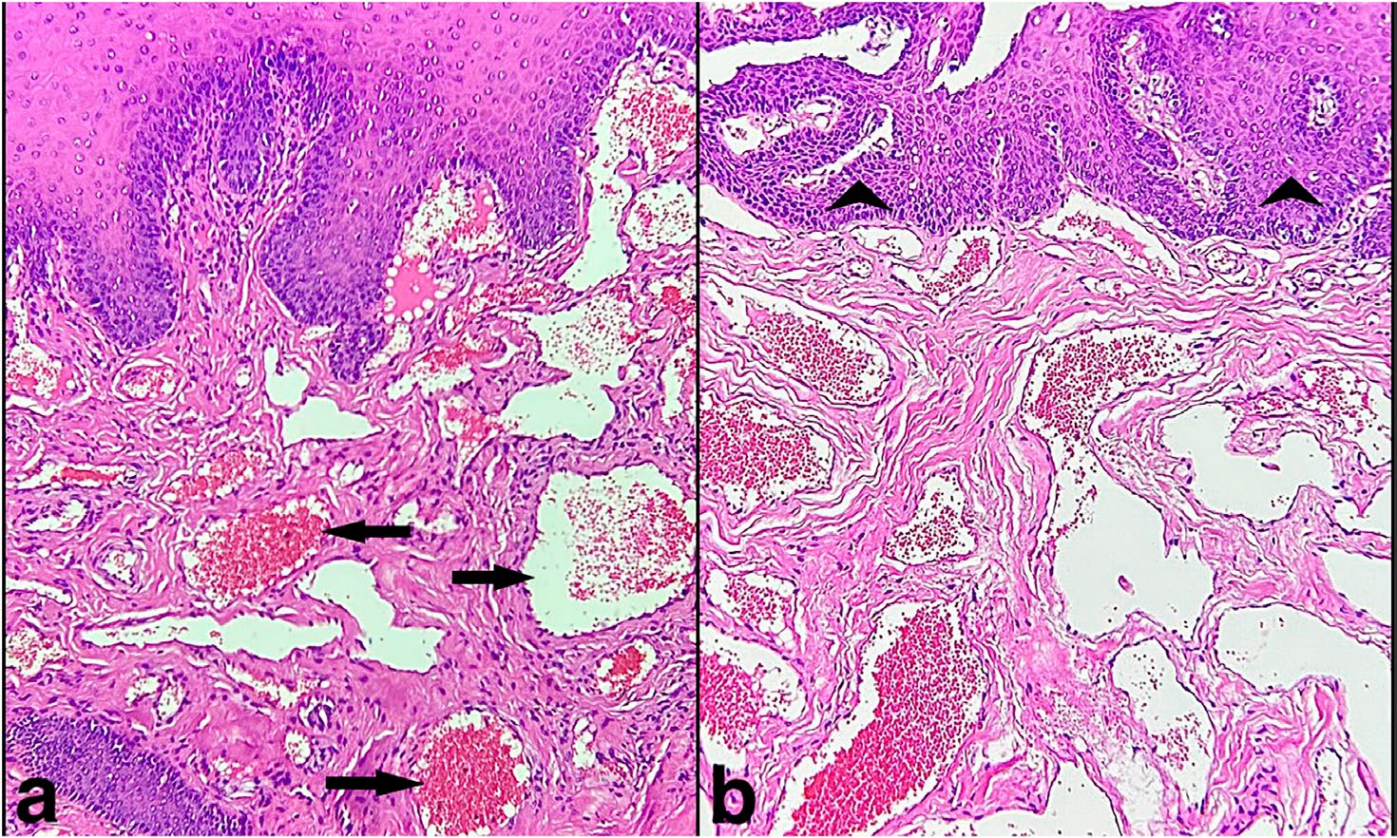
Histopathologic sections showing (a) multiple thin‐walled vascular spaces containing erythrocytes (arrows), located subjacent to (b) the tongue epithelium (arrowheads) (H&E, ×400).

## 3 Differential Diagnosis, Investigations, and Treatment

The clinical presentation of recurrent bleeding oral papules prompted a systematic differential diagnosis including hereditary benign telangiectasia, CREST syndrome, and mucosal telangiectasias of aging. Investigations included a detailed medical and family history, with specific inquiry into epistaxis frequency, pulmonary involvement, and known genetic diagnoses among first‐degree relatives. Physical examination was also conducted, and diascopy was performed on the lingual lesions. In addition, a panel of laboratory investigations was ordered, including hemoglobin (Hgb), hematocrit (HCT), red blood cells (RBC), white blood cells (WBC), platelets (Plt), and ferritin. Following surgical excision of representative lingual lesions, tissue specimens were submitted for histopathological examination. Routine hematoxylin and eosin (H&E) staining was performed to evaluate vascular architecture. Surgical intervention was planned for symptomatic control of bleeding.

## 4 Outcome and Follow‐Up

The initial surgical intervention resulted in immediate cessation of bleeding from the treated lingual lesions. The postoperative course was uneventful, with no wound infection or delayed healing. At the one‐year follow‐up visit, the patient reported recurrence of the same chief complaint, with reappearance of bleeding lingual lesions. This recurrence reflects the systemic nature of the vascular alterations underlying HHT; therefore, local surgical management serves only to relieve symptoms and does not address the generalized vascular disorder.

## 5 Discussion

This report describes a rare case of lingual telangiectasias in a 54‐year‐old female with HHT. Telangiectatic lesions in HHT typically appear during the third and fourth decades of life and increase in number with age [3, 4], consistent with the initial identification of HHT in the present patient at the age of 24 years.

Mucocutaneous telangiectasias of the tongue, lips, palate, or fingers are reported in 50 to 80% of HHT patients [2]. Hyldahl et al. [4] noted that men tend to develop more lesions on the forehead, nose, and hands than women, a difference possibly attributable to physical activity and sun exposure [4]. A distinctive feature of the present case is the isolated involvement of the tongue, with no other oral mucosal sites affected.

Epistaxis is the most common clinical presentation, occurring in over 90% of patients [1, 2, 7], which aligns with the history obtained in this case. Bowers et al. [2] emphasized that although nasal bleeding is a key indicator of the disease, lingual involvement can also lead to substantial complications. Accordingly, the chief complaint of our patient was severe bleeding from tongue lesions, rather than the coexisting epistaxis. The most severe bleeding episodes are observed in patients older than 40 years [2], which is consistent with our patient's report of worsening bleeding in recent years.

The involvement of the patient's sister and uncle, despite the absence of disease in her children, is compatible with the autosomal dominant inheritance pattern of HHT. It is estimated that approximately half of the offspring of an affected parent will inherit the condition, with variable expressivity [5].

Clinically, Hyldahl et al. [4] described telangiectatic lesions as macular (flat) or more papular (elevated), with a round morphology being more common than linear forms; radicular, tortuous, or simply dilated capillaries may also be observed. In the current patient, the lingual lesions presented as papules.

The management of oral HHT is not well established, with most reports focusing on the treatment of epistaxis [2, 3]. In the present case, given the progressive nature of the disease and its high recurrence rate, surgical intervention served solely as symptomatic treatment for lingual bleeding. Asymptomatic telangiectasias do not require treatment [3]. Initial management of lesions includes diet modification and topical therapies such as ointments, saline sprays, and topical hemostatic agents [2]. When further intervention becomes necessary, various modalities have been described, including laser ablation alone or in combination with bevacizumab (Avastin), sclerotherapy, and embolization [3]. Systemic therapies such as tamoxifen and tranexamic acid have been employed for vascular lesions, although both carry an increased risk of venous thromboembolism. Recalcitrant telangiectasias may be managed with coblation, bipolar cautery, or laser photocoagulation, among other techniques. Potassium titanyl phosphate (KTP) laser has been proposed as a suitable treatment modality for tongue telangiectasias. Bevacizumab, a monoclonal antibody that binds vascular endothelial growth factor (VEGF) and inhibits angiogenesis, has shown increased efficacy when administered via mucosal injection in combination with surgery. Surgical excision remains a rational option for deep vascular lesions [2].

During follow‐up, particular attention should be directed to the lungs and brain, as both may harbor clinically silent lesions capable of causing sudden death [7]. As demonstrated in this case, antibiotic prophylaxis—similar to the regimen recommended for infective endocarditis—is warranted in dental procedures because of the risk of bacteremia in patients with pulmonary involvement [8]. Several reports have documented brain abscesses following dental procedures in HHT patients [3]. To the best of the authors' knowledge, this represents the most critical consideration regarding HHT for dentists and oral and maxillofacial surgeons, who may serve as the first identifiers of this syndrome. Notably, timely diagnosis of HHT is essential for the appropriate management of its complications [1].

## 6 Conclusions

Lingual telangiectatic papules may represent an oral manifestation of HHT and can cause recurrent bleeding that prompts patients to seek professional healthcare. Dental practitioners may play a pivotal role in the early recognition of this disease, as they may be the first to identify its oral manifestations. Early identification is essential to ensure appropriate dental management, including consideration of antibiotic prophylaxis before invasive dental procedures that pose a risk of bacteremia in patients with pulmonary involvement.

## Author Contributions

**Saede Atarbashi‐Moghadam:** conceptualization, investigation, supervision, visualization, writing – review and editing. **Ali Lotfi:** methodology, investigation, validation, visualization. **Fazele Atarbashi‐Moghadam:** visualization, supervision. **Mostafa Alam:** supervision, writing – review and editing. **Milad Baseri:** methodology, writing – original draft, writing – review and editing, project administration. **Mohammadreza Kashefi Baher:** conceptualization, investigation, visualization, data curation, formal analysis, methodology, writing – original draft, writing – review and editing.

## Funding

The authors have nothing to report.

## Ethics Statement

The authors have nothing to report.

## Consent

Written informed consent for the publication of this case, including all clinical details and any accompanying images, was obtained from the patient.

## Conflicts of Interest

The authors declare no conflicts of interest.

## Supporting information


**Supplementary Table 1** Preoperative laboratory values.

## Data Availability

Data sharing not applicable to this article as no datasets were generated or analysed during the current study.
