# Supplementary material for: Hereditary Hemorrhagic Telangiectasia of the Tongue: A Rare Case Report
Source: Clin Case Rep. 2026 Nov 6;14(11):e73883. doi: 10.1002/ccr3.73883 (PMC13741848; doi:10.1002/ccr3.73883)
Supplement: Supplementary file 1 [file CCR3-14-e73883-s001.docx]

| **Supplementary Table 1. Preoperative laboratory values** | | |
| --- | --- | --- |
| **Parameter** | **First Surgery** | **Second Surgery** |
| Hemoglobin (Hgb, g/dL) | 8.2 | 9.8 |
| Hematocrit (Hct, %) | 27.7 | 31.0 |
| Red blood cell count (RBC, ×10^6^/µL) | 4.0 | 3.9 |
| White blood cell count (WBC, ×10^6^/µL) | 5.39 | 6.2 |
| Platelet count (Plt, ×10^3^/µL) | 359 | 280 |
| Ferritin (ng/mL) | 5.59 | 11.27 |
